# Neural Discrimination of Temporal Patterns—Associations to Dyslexia Risk, Language Abilities, and Music Activities

**DOI:** 10.1111/nyas.70101

**Published:** 2025-10-15

**Authors:** Paula Virtala, Sergio Navarrete‐Arroyo, Eino Partanen, Minna Huotilainen, Teija Kujala

**Affiliations:** ^1^ Finnish Centre of Excellence in Music, Mind, Body, and Brain, Cognitive Brain Research Unit, Department of Psychology, Faculty of Medicine University of Helsinki Helsinki Finland; ^2^ Finnish Centre of Excellence in Music, Mind, Body, and Brain, Cognitive Brain Research Unit, Faculty of Educational Sciences University of Helsinki Helsinki Finland

**Keywords:** dyslexia, ERPs, event‐related potentials, language skills, mismatch negativity, music activities, temporal auditory processing

## Abstract

Developmental dyslexia is associated with deficient temporal auditory processing, which may play an important role in speech perception, language development, and subsequently, learning to read. Music activities may offer a way to support temporal auditory processing and language and reading development. We utilized change‐related mismatch negativities (MMNs) of the electroencephalogram (EEG) to study the detection of temporal pattern violations in tone sequences in 28‐month‐olds, and its associations with dyslexia risk, language skills, and music activities. We also considered possible effects of two infant music‐listening interventions. The MMN had diminished amplitudes in dyslexia risk compared to control children in the left hemisphere, and left‐hemisphere dominance was only seen in the controls. Larger MMNs were associated with better language skills and higher amount of parent‐reported shared music activities, but not with the infant music‐listening interventions. The results demonstrate, in line with our hypotheses, deficient processing of temporal patterns in tone sequences in familial dyslexia‐risk children. Together with the positive association with language skills, this supports the relevance of temporal auditory processing for language and reading development. The association of MMNs with the frequency of shared music activities at home suggests that active, joint music making might benefit temporal auditory processing.

## Graphical abstract

This mismatch negativity (MMN) study showed deficient processing of temporal patterns in tone sequences in dyslexia‐risk children aged 28 months. The MMN magnitude to temporal pattern violations showed positive associations with language skills. Results support the proposed role of auditory temporal processing deficits in dyslexia. Music activities might support auditory temporal processing, as the frequency of music activities at home was associated with larger MMNs.

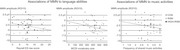

## 1 Introduction

Developmental dyslexia is a prevalent, heritable reading deficit that is associated with difficulties in speech processing and language development that can already be detected in infancy and early childhood and are largely understood to stem from a phonological deficit [1]. However, the auditory processing deficits in dyslexia may extend beyond speech processing to broader, underlying auditory abilities that may cause or contribute to the phonological deficit. Among these abilities, several influential scholars have suggested deficient temporal processing to be a core deficit underlying dyslexia [2, 3, 4, 5, 6, 7] or even neurodevelopmental disorders more generally [8]. However, to show that a temporal processing deficit is causally linked to the development of dyslexia, it should be investigated in young at‐risk children before the onset of reading and reading instruction [9, 10].

For investigating auditory processing in young age groups, event‐related potentials (ERPs), and among them, the change‐related mismatch negativity (MMN; often referred to as mismatch response, MMR, in infants and young children), are feasible measures. MMN has been used to study neural auditory processing deficits in dyslexia and its familial risk already at birth (for reviews see Refs. [11, 12, 13]; for a recent meta‐analysis see Ref. [14]). In several longitudinal datasets, neural auditory processing has been atypical in at‐risk infants and small children in speech and nonspeech contexts [15, 16, 17, 18, 19, 20, 21, 22] and also associated with subsequent language and reading abilities [22, 23, 24, 25]. Many of these deficits have been interpreted to reflect atypical temporal processing (see below). A recent meta‐analysis proposed that in the nonspeech domain, a specific type of temporal auditory processing, namely, discrimination of temporal patterns in tone sequences, is impaired in adults with dyslexia and deserves further investigation in child populations [14]. The present study aimed to investigate its role in the development of dyslexia in early childhood. To this end, MMNs to temporal structure violations in tone sequences were recorded from ∼2‐year‐old children, and cross‐sectional associations to both familial dyslexia risk and language outcomes were examined.

As infants and children at risk for dyslexia show auditory and temporal processing deficits that are associated with future language and literacy, auditory and temporal processing offers a promising target for early preventive interventions. The beneficial role of music activities for language and literacy is widely established [26]. It is generally understood to be driven by music's benefits for auditory processing [26], or specifically, auditory temporal (or rhythm) processing [27, 28]. In infants and young children, music and especially music making in social settings have generally been associated with improved auditory processing and language development (reviewed in Ref. [29]). However, there is less research on its associations with temporal auditory processing. The present study aimed to investigate this by determining whether informal musical activities in infancy and early childhood are associated with improved temporal auditory processing in childhood.

### 1.1 Auditory and Temporal Processing Deficits in Dyslexia and Its Familial Risk

Various deficits in nonspeech auditory processing have been identified in dyslexia, although some of them seem to be more prevalent and consistent than others. A systematic review focusing on nonspeech auditory processing deficits in children and adults with dyslexia identified problems in discrimination of sound durations and small changes in sound frequency, discrimination of rise time, as well as in processing high amplitude modulations and slow frequency modulations [11]. In contrast with these results, a more recent meta‐analysis focusing specifically on MMN studies in this field identified nonspeech processing deficits only in adult with dyslexia, and mainly only in the processing of temporal patterns in tone sequences with no such studies in children with dyslexia [14] (see also Ref. [12]). However, these previous reports left out research on infants and small children at risk for dyslexia. Younger age groups may have deficits that are no longer seen in older individuals due to, for example, maturational or compensatory mechanisms. These deficits may still contribute to the development of dyslexia.

Brain research already has shown various auditory processing deficits in infants as risk for dyslexia, but a large part of it focuses on speech processing. For example, MMRs have been atypical in at‐risk infants in response to speech sound duration changes [15, 16, 17] (see also Refs. [30, 31]) as well as vowel [17] and consonant changes in syllables [18, 19] (see also Ref. [32]). In several longitudinal datasets, these deficits were also associated with subsequent problems in reading and related skills, suggesting that auditory processing skills contribute to their development [22, 23, 24, 33].

In the nonspeech domain, in infants at family risk for either dyslexia or developmental language disorder, neural processing of duration and frequency changes in rapidly presented tone pairs was deficient and longitudinally associated with expressive vocabulary in early childhood [20] (see also Refs. [34, 35]). These results have been interpreted in light of the so‐called rapid auditory processing deficit theory [3]. According to this framework, both dyslexia and developmental language disorder (previously specific language impairment) would be characterized by deficient processing of brief, rapidly successive acoustic changes [3]. Thus, also speech processing problems such as deficient consonant discrimination could be explained by problems in (temporal) processing of brief formant transitions in these phonemes [3].

Another influential view for the auditory deficits in dyslexia not restricted to speech processing is described in the temporal sampling framework that specifically highlights difficulties in people with dyslexia in processing amplitude rise time and suggests a neural‐level explanation for them, namely, poor alignment of neuronal oscillations with amplitude modulations in the speech signal [5]. Dyslexia has been associated with deficits in processing amplitude rise time that acts as a temporal cue in speech segmentation [36], but neural‐level evidence in at‐risk infants and young children remains limited [10]. On a behavioral level, amplitude rise time sensitivity in infancy has been found to predict vocabulary development (although it was not associated with dyslexia risk) [37]. Furthermore, an MMN study demonstrated problems in both frequency and amplitude rise time discrimination in a nonspeech context in ∼3‐year‐old children at risk for dyslexia, with predictive associations with future reading abilities [21] (see also Ref. [38]).

Auditory cortical areas are understood to have a division of labor, with the left hemisphere specializing in rapid and right hemisphere in slow changes [39, 40]. Accordingly, the left hemisphere has an important role in speech processing and its dysfunctions in dyslexia [1], and several studies have shown atypically right‐lateralized or bilateral processing of speech in dyslexia risk [15, 41] as well as positive associations of left‐lateralized speech sound processing in infants with subsequent language and literacy issues [24, 25]. However, right‐hemisphere activity is also important for speech processing on slow temporal rates such as amplitude rise time and prosody, and should thus play a role in the development of dyslexia, as postulated in the temporal sampling framework [5] (see also Ref. [42]). Also in the above‐reviewed MMN/MMR studies, deficient rapid auditory processing was reported in infants at risk for dyslexia and developmental language disorder specifically in the right hemisphere [20]. To increase the understanding of the role of the two hemispheres in auditory processing deficits underlying dyslexia, more research is needed, especially in the developing brain.

### 1.2 Temporal Pattern Processing in Dyslexia and Dyslexia Risk

One relevant but less studied way to investigate auditory temporal processing in dyslexia is to modify the temporal pattern of tone sequences, as brought up in a recent review [14]. Studies on processing temporal pattern violations in tone sequences in dyslexia have used tones and changes in their timing. For example, four‐tone and two‐tone sequences were presented to adults with or without dyslexia, and a group difference was observed in the MMN elicited only by the more complex four‐tone sequence [43] (see also Ref. [44]). The temporal pattern violation was caused by a too‐early third tone in the sequence. In an ignore condition with participants’ attention directed away from the sounds, an early MMN to the too‐early third tone was absent in adults with dyslexia, while a later MMN to the tone omission (at the expected time of the third tone) was obtained in both participants with dyslexia and typically reading participants. Interestingly, the later MMN was right‐lateralized only in typical readers, suggesting atypical (or absent) hemispheric lateralization in the participants with dyslexia in processing temporal pattern of tone sequences. In a following behavioral task where participants were instructed to detect any deviant stimuli, adults with dyslexia also had lower hit‐rates than controls for the temporal violations [43].

One longitudinal study investigated similar temporal pattern processing in tone sequences in dyslexia‐risk infants [22]. The stimulus paradigm differed from the above‐introduced paradigm [43] only in interstimulus interval [22]. At 17 months, only control children exhibited MMRs to the temporal pattern violation in the tone sequence (a negative MMN followed by a positive MMR), while the dyslexia‐risk children showed no statistically significant MMRs. The positive MMR amplitude was positively associated with both language comprehension scores at 53 months and reading fluency scores at second grade [22]. The only other somewhat similar study that the authors are aware of compared small groups of dyslexic and typically reading Chinese school children [45]. A nonsignificant trend of diminished MMNs was found in children with dyslexia in response to a temporal pattern violation in a three‐tone sequence, where timing of the middle tone was manipulated. Together these somewhat scattered findings in small samples (only 8–12 per group) suggest that a temporal tone sequence processing deficit may be evident in dyslexia and dyslexia risk as absent, diminished, or atypically lateralized MMNs/MMRs. More research is needed to confirm the deficit and how it is manifested in the MMNs/MMRs in different age groups, including their hemispheric lateralization that has not been studied in children.

### 1.3 Promises of Music for Auditory Temporal Processing, Language, and Reading Abilities

Temporal processing deficits in dyslexia and related speech and language disorders were recently suggested to stem from atypical rhythm, which was highlighted as a potentially important risk factor for these disorders (i.e., the Atypical Rhythm Risk Hypothesis) [7]. This hypothesis emphasizes the considerable overlap between musical rhythm and speech/language processing, promoting also the relevance of music‐based intervention opportunities for dyslexia and related disorders.

Indeed, a notable research literature supports the role of temporal auditory processing in the positive association of music activities with language and reading abilities. First, interventions specifically targeting auditory temporal processing improve language and reading. A randomized controlled trial (RCT) suggested that a music intervention focusing on rhythmic aspects was more beneficial for reading and related skills in dyslexic children than a painting intervention [46]. Rhythm‐based auditory interventions have also shown comparable effects on reading‐related measures in dyslexic children than interventions that directly target phoneme discrimination [47] or grapheme–phoneme matching [48]. Second, music activities show positive associations with auditory temporal processing. In the aforementioned RCT, rhythm reproduction skills improved with the music intervention (these skills were also specifically trained in the intervention) [46]. One previous study in infants demonstrated benefits of a social music group focusing on the triple meter (waltz) for processing temporal structure in both speech and music [49]. These kinds of benefits should be investigated also in infants and young children at risk of dyslexia. Taken the predictive role of early auditory processing for subsequent language and literacy (reviewed above), musical activities that promote auditory processing in at‐risk infants and children could be recommended as preventive measures for future reading problems.

To this end, as part of the DyslexiaBaby study that the present study also belongs to, a music‐listening intervention study was conducted to examine the possibilities to support neural speech processing and language development with vocal music exposure in infants at dyslexia risk [29, 50]. In this intervention study, dyslexia‐risk infants were pseudorandomly assigned to a vocal music‐listening group or a control intervention—an instrumental music‐listening group—both consisting of regular (∼1 h/week) music exposure at home for the first 6 months of life. The study also included a *silent* no‐intervention group. Results from the intervention study showed that in line with the hypotheses, the vocal but not the instrumental music‐listening intervention improved neural speech sound discrimination in dyslexia‐risk infants, as reflected by the MMRs [50].

This benefit for speech sound processing after vocal music listening was seen immediately after the intervention at the age of 6 months but it was no longer significant at 28 months of age [50]. In addition to the long follow‐up period, the lack of an effect at 28 months was possibly attributable to the passive nature of the intervention. Indeed, while passive music exposure has been shown to facilitate neural auditory processing also in some previous studies [51], active, social music making seems to promote early auditory processing and language learning better than passive exposure [34, 52, 53]. If auditory temporal processing is linked with musical activities in early age, and at the same time associated with dyslexia risk and language development, this would support its role in mediating the benefits of music for language development. However, according to our knowledge, previous research on the association of informal early musical activities with auditory temporal processing in healthy and at‐risk populations is largely lacking.

### 1.4 Study Objectives and Research Questions

With already a considerable amount of previous research on the role of early speech processing deficits in dyslexia risk and their associations to language and literacy, nonspeech processing deficits have remained less studied and many previous studies in this field combined children with parental dyslexia and developmental language disorder (see above). While temporal auditory processing has been approached from several different viewpoints such as rapid auditory processing and amplitude rise time detection (see above), the temporal pattern of tone sequences is a rarely studied aspect that can catch some of the temporal complexities of continuous speech while still being separate from, for example, phonemic processing. Furthermore, previous studies investigating the associations of early childhood musical activities with auditory and language abilities have not examined temporal auditory processing specifically.

Thus, in the present study, neural detection of temporal pattern violations in tone sequences was studied in young children as an example of a nonspeech auditory temporal processing deficit. To achieve this, we presented a similar tone sequence MMN paradigm to that by Kujala et al. [43] and van Zuijen et al. [22] to 28‐month‐old children, a cross‐sectional subsample of the DyslexiaBaby longitudinal study, where ∼3/4 of the children have a familial risk for dyslexia. Approximately at the same age, we also collected information on language development with standardized tests and questionnaires and on early childhood musical activities with custom‐made parental questionnaires. Part of the infants had additionally participated in one of two infant music‐listening interventions between birth and 6 months.

We studied how the tone sequence MMNs and their hemispheric lateralization are associated with familial dyslexia risk, standardized measures of language development, and early musical activities. We expected to find absent, diminished, or atypically lateralized MMRs/MMNs in the dyslexia‐risk children, with positive associations between MMN/MMR magnitude and language abilities. We also expected to see positive associations between the MMN/MMR magnitude and parent‐reported social and active music activities, specifically, musical playschool attendance and shared music activities at home in early childhood. In contrast, we expected no or minor effects of an infant music‐listening intervention on temporal auditory processing at the age of 28 months due to its passive nature, but nevertheless took the possible intervention effects into account in the analyses. Similarly, we also expected no or minor associations between the MMN/MMR magnitude and more recent, parent‐reported amount of passive music exposure/music listening. The results of the present study can contribute to the discussion on the role and width of nonspeech and temporal processing deficits in dyslexia (risk), their relevance for early language development, and the links between early musical activities with early auditory abilities.

## 2 Methods

### 2.1 Participants

Participants of the present study consist of those children of the DyslexiaBaby longitudinal sample who were presented with the tone sequence paradigm in an EEG recording at the age of 28 moths. The tone sequence paradigm results reported here were only obtained cross‐sectionally in this age group and only approximately from half of the total DyslexiaBaby sample. The tone sequence experiment was conducted on those children who were not too tired, fussy or hungry to continue the EEG experiment for approximately 20 min (after the presentation of other paradigms, see Section 2.2). The DyslexiaBaby longitudinal study, including this study, has been approved by the Ethics Committee for Gynaecology and Obstetrics, Pediatrics and Psychiatry of the Hospital District of Helsinki and Uusimaa, and was conducted in compliance with the Declaration of Helsinki. One or both caregivers signed a written informed consent when their child was enrolled to the study at birth.

The present participant sample consists of children with (risk group) or without (control group) familial risk for developmental dyslexia, all with at least one parent who is a native Finnish speaker. To be included in the risk group, the children had to have at least one biological parent with confirmed (recent diagnostic statement or reading tests in the study) or compensated dyslexia (see below). The reading tests consisted of four subtests of a Finnish standardized test [55]: reading a narrative text, a word list, and a pseudoword list and writing words. Dyslexia was confirmed when (1) speed or accuracy was at least one standard deviation (SD) below the norm in at least two subtests and (2) the parent reported clear reading and writing deficits in childhood. When only the second criterion was met, dyslexia was deemed compensated if the parent additionally reported dyslexia in close biological relatives. Exclusion criteria were neurodevelopmental conditions other than dyslexia/developmental language disorder as well as brain trauma during childhood in the parents.

All the risk group children in the DyslexiaBaby longitudinal study, including all the risk group children in the present study, had been pseudorandomized to three intervention subgroups at birth, one receiving a vocal music‐listening intervention in infancy (VOC group), one receiving an instrumental music‐listening intervention at the same age (INS group), and one receiving no intervention (NO‐INT group, see Table 1). For details on the interventions, see Section 2.1.1. and Refs. [29, 50]. To be included in the control group (CON), one (if the other one was not available) or both biological parents of the child had to report no suspected or diagnosed learning‐ or language‐related deficits including developmental dyslexia.

**TABLE 1:** Background information in the total sample included in the EEG analyses.

	VOC (*N* = 19)	INS (*N* = 20)	NO‐INT (*N* = 23)	CON (*N* = 16)	HIGH‐RISK (*N* = 43)
Girls/boys	8/11	11/9	11/12	10/6	17/26
High/low education	18/1	18/2	20/2	15/1	38/4
Age, months (SD)	28.1 (0.5)	28.1 (0.4)	28.2 (0.4)	28.3 (0.4)	28.1 (0.4)

*Note*: The columns describe background information in the risk groups with the vocal (VOC) or instrumental music‐listening intervention (INS) and without intervention (NO‐INT), and in the control group (CON), as well as in the combined risk group (HIGH‐RISK) that was compared against the CON group. The HIGH‐RISK group consists of the VOC, INS, and NO‐INT groups, with those children excluded whose parents were deemed compensated dyslexics.

In univariate ANOVAs, measurement age did not differ statistically significantly between the four groups VOC, INS, NO‐INT, and CON (*p* > 0.20) or between the HIGH‐RISK and CON groups (*p* = 0.158). Gender distribution and the amount of high/low education (low education refers to no higher level/tertiary education in either of the caretakers) among caretakers did not differ statistically significantly between the four groups or the HIGH‐RISK and CON groups in Pearson Chi‐square tests (*p* > 0.20, except for gender distribution between HIGH‐RISK and CON groups, *p* = 0.115). Education information was missing from one child in the NO‐INT group (included also in the HIGH‐RISK group).

MMNs elicited by the tone sequence paradigm were recorded from a total of 97 participants at 28 months out of whom a total of 19 participants were excluded from all analyses due to the following reasons: four were excluded due to reasons related to parental diagnosis that came up after enrolment to the study (failure to meet the risk group inclusion criteria), five due to failure to follow through with the infant music‐listening intervention (two in the vocal, three in the instrumental intervention group; for details see below and Ref. [50]), and 10 due to problems in EEG data quality (see below). This resulted in a total *N* = 78 (total sample with all risk group children and control children; see Table 1 for details).

In line with previous publications from the project [17], the effect of dyslexia risk on the MMNs was analyzed from the risk group children whose parental dyslexia was confirmed, while the risk group children with compensated parental dyslexia were excluded from these analyses (this remaining group was termed HIGH‐RISK and it included the risk group children with confirmed parental dyslexia from all the three intervention subgroups; see Table 1). Group comparisons of the MMNs were conducted first to the three intervention subgroups to study the effects of the infant music‐listening interventions (VOC, INS, and NO‐INT) and then for the high‐risk and control groups to study the effect of dyslexia risk (HIGH‐RISK and CON). For samples included in the statistical analysis of the associations of the MMNs with language test scores, parental language questionnaire scores, and parent‐reported musical activities, see Table 2.

**TABLE 2:** Scores, sample sizes, and background information of the participants included in the statistical analysis of the associations of the MMNs with language test scores.

Studied association	Score (SD)	Sample size (VOC/INS/NO‐INT/CON)	Girls/boys	High/low education	Age, months (SD)
**Reynell**		78 (19/20/23/16)	40/38	71/6*	28.2 (0.4)
CS raw score	30.4 (8.6)				
ES raw score	11.2 (5.7)				
**MCDI**		64 (15/17/18/14)	33/31	58/6	28.1 (0.4)
Vocab. size, word count	375.5 (154.1)				
MLU, morpheme count	9.4 (4.1)				
**Musical activities**		69** (16/18/22/13)	34/35	65/4	28.1 (0.4)
Shared music, 1–4	3.1 (0.6)				
Mus. playsch. att. (yes/no)	43/26				
Music exposure, h/week	8.6 (8.5)				

*Note*: The scores are extracted from the Comprehension Scale, CS, and Expressive scale, ES, of the Reynell Developmental Language Scale III [62], from the standardized parental questionnaire MacArthur‐Bates Communicative Development Inventory (MCDI) [63], providing vocabulary size and mean length of utterance, MLU), and from a parental questionnaire on the child's musical activities, including Shared music, Musical playschool attendance, and Music exposure.

*For one participant, parental education information was not available. **For two participants, information on shared music activities and music exposure was not available. Low education refers to no higher level/tertiary education in either of the caretakers. MCDI was missing (not returned on time) from 14 participants of the total sample in Table 1. The parental questionnaire on musical activities of the child was missing (not returned on time) from nine participants of the total sample in Table 1. For all studied associations, a laterality index describing hemispheric lateralization of the MMNs could not be quantified from *N* = 7 children. Thus, sample sizes for those associations should be subtracted by seven. For all studied associations, MMN peak latency could not be quantified from *N* = 10 participants. Thus, sample sizes for those associations should be subtracted by 10. Abbreviations: CON, control group; CS, comprehension scale; ES, expressive scale, INS, instrumental music‐listening intervention group; MCDI, MacArthur–Bates Communicative Development Inventory; MLU, mean length of utterance; NO‐INT, no intervention group; VOC, vocal music‐listening intervention group.

#### 2.1.1 Infant Music‐Listening Interventions

The infant music‐listening interventions were administered to two dyslexia risk subgroups between birth and 6 months of age. They consisted of playing music to the infants via a custom‐designed online song library including Finnish children's and folk songs in 20‐min playlists, with a tablet computer and a portable loudspeaker. The parents had been instructed to play at least five song lists per week in peaceful surroundings and without singing along. The melody was sung by a vocalist (VOC group, with Finnish lyrics and each song with one of two female or three male singers) or played by an instrument (INS group, each song with one instrument: banjo, mandolin, xylophone, or metallophone) and always accompanied by soft acoustic guitar. A successful intervention lasted at least ∼5 months with an approximate minimum intensity of ∼1 h/week (for at least 4 months) and at least 24 h of listening time in total.

### 2.2 Experimental Stimuli and Paradigm

The experimental stimuli and paradigm were designed based on Kujala et al. [43]. However, instead of sinusoidal tones, the stimuli were naturally produced and recorded claves tones in order to create a more pleasant and natural auditory environment, and the interstimulus interval was shortened in order to reduce the recording time due to the young age of the participants. The sounds were modified with Audacity software (2.3.2) to reduce the attack by adding a rise and fall time of ∼3 ms to make the sound 30 ms long.

**FIGURE 1:**
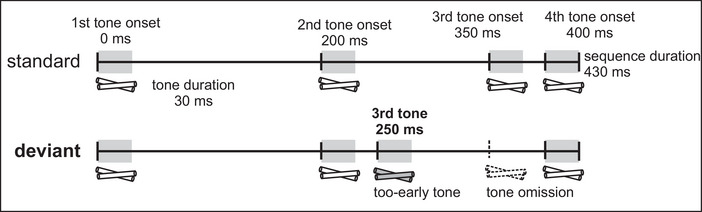
Standard and deviant sequence in the tone sequence paradigm. The horizontal line illustrates the duration of the tone sequence, with duration of the stimuli (clave tones) illustrated with the gray bars. The clave symbols behind the horizontal line indicate the individual clave tones, and the difference between the sequences is marked in the deviant sequence with a gray (too‐early tone) and a dashed claves symbol (missing tone).

Individual sounds were composed into one standard and one deviant sequence, both with four sounds of 30 ms each and silent breaks between the sounds as follows. In the standard sequence, sound onsets were at 0 ms, 200 ms, 350 ms, and 400 ms from sequence onset, while in the deviant sequence they were at 0 ms, 200 ms, 250 ms, and 400 ms from sequence onset. This resulted in a total duration of 430 ms for both sequences (Figure 1). The experiment included three blocks of 399 stimuli, with 60 deviants (15%) and 339 standards (85%) presented with a 900‐ms interstimulus interval, resulting in a total of 180 deviants and a total duration of ∼18 min.

### 2.3 EEG Recording

**FIGURE 2:**
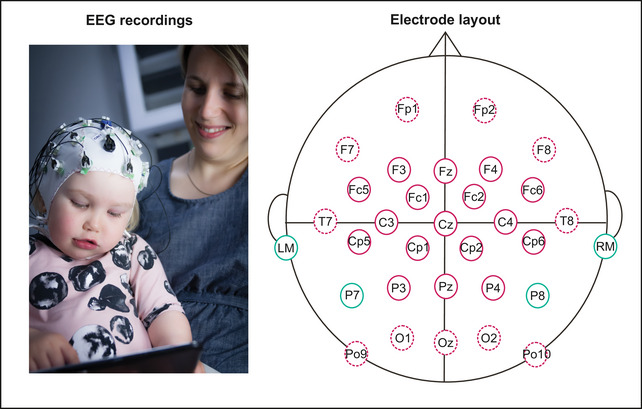
Left: A photo illustrating the EEG recording at 28 months in the DyslexiaBaby study. The photo is taken by Anastasia Gallen, with permission from both parents of the child to use the photo. Right: Electrode layout in the EEG recording, with peripheral electrodes indicated with dashed circles and reference electrodes with green circles.

The EEG was recorded with a BrainProducts QuickAmp amplifier (v. 10.08.14; software: BrainVision Recorder 1.20.0801, Brain Products GmbH, Gilching, Germany; sampling rate 500 Hz, low‐pass filter 100 Hz, high‐pass filter 0 Hz, average reference) and Acticap 32‐electrode cap (Brain Products GmbH, Gilching, Germany; see Figure 2). Presentation 17.2 Software (Neurobehavioural Systems Ltd., Berkeley, CA, USA) and two Genelec speakers (∼65 dB sound pressure level) were used for paradigm presentation. The recording including preparations took approximately 2 h and included an additional experimental paradigm presented before the present paradigm (with speech stimuli, reported elsewhere [17, 50, 55]).

The EEG recording was conducted in a sound‐proof and electrically shielded laboratory booth of the University of Helsinki (*N* = 73) or University of Jyväskylä (*N* = 5), with the child awake, sitting on a chair (distance from the speakers 160 cm) and playing silenced tablet games or watching silenced cartoons. The child was always accompanied by a caregiver or research assistant or sat on their lap and was advised to stay still and silent and to ignore the sounds presented. The child's willingness to participate was respected as much as possible and an illustrated leaflet of the EEG recording was mailed to the families beforehand to reduce fear and ensure informed assent of the children.

### 2.4 EEG Data Analysis

Preprocessing (Matlab 2017a–2020a, The MathWorks, Inc., USA; EEGLAB 14.0.0b [57] and 2019_0 [57]; ERPLAB 7.0.0 [58]) included seven main steps. After preliminary filtering (step 1; 0.025–40 Hz band pass), poor‐quality electrodes (maximum of six per block, with a larger number of bad electrodes resulting in omitting the whole data block from analysis; peripheral ones omitted from the data; nonperipheral ones marked down for step 5) and data segments (omitted from the data) were searched based on visual inspection (step 2). The signal of a certain electrode was deemed of poor quality when it was either flat or dominated by high‐frequency noise or continuous large drifts. A data segment was deemed of poor quality when both the continuous signal and recording notes time‐stamped to the data file indicated muscle‐related artifacts across electrodes. The original data were filtered 0.5 Hz high pass and 25 Hz low pass (step 3) and re‐referenced to the average signal of four ear‐adjacent electrodes (step 4; LM, RM, P7, P8; Figure 2). Then nonperipheral electrodes with poor quality were interpolated (step 5, based on step 2; F3, Fz, F4, C3, Cz, C4, P3, Pz, P4, FC5, FC1, FC2, FC6, CP5, CP2, CP3, CP6; maximum three electrodes per participant; not adjacent electrodes) and independent component analysis (fastica [59] or runica, EEGLAB) was performed to correct for eye‐movement and heart beat artifacts (step 6; only if the components were correctly identified based on visual inspection).

Finally, the data were epoched (step 7; −100 to 840 ms; −100 to 0 ms baseline correction) and epochs were omitted if the amplitude exceeded ± 120 µV at Fp1 and Fp2 or they contained a drift of > 100 µV or included data points ± 3 SD from the mean amplitude of all epochs (jointprob in EEGLAB). The standard stimulus epochs immediately following a deviant stimulus were omitted. Epochs were merged leaving one standard and one deviant dataset per participant; less than 30 deviant epochs in a dataset resulted in exclusion of that participant from the analyses (*N* = 7). Additionally, data of three participants were excluded before the preprocessing steps due to technical problems, very poor data quality, or the child accidentally falling asleep during the recording. These 10 participants were not included in the statistical analyses as described above in the Participants section. This resulted in an average of 88 (range 34–139) deviant epochs per child in the final dataset (with means of 90, 92, 82, 91, and 89 in the VOC, INS, NO‐INT, CON, and HIGH‐RISK groups, respectively; one‐way analyses of variance [ANOVA] for the VOC vs. INS vs. NO‐INT groups and for the HIGH‐RISK vs. CON groups yielded no statistically significant group differences, for both *p* > 0.20). Standard and deviant responses were formed by averaging together the signal across the respective epochs. For MMN quantification, standard responses were subtracted from deviant responses.

In order to determine significant differences between the standard and deviant responses, cluster‐based mass permutation tests (Fieldtrip toolbox [59, 60]) were employed to all nonperipheral electrodes (not reference electrodes) data point by data point in the whole sample. Those time ranges that showed statistically significant (*p* < 0.05) deviant‐standard differences (with same polarity in adjacent points and at min two neighboring electrodes), for each such significant cluster, the sum of *t* values was calculated and the test statistic defined as their maximum. Five thousand random permutations of the deviant versus standard, with test statistic computed for each iteration, were used to determine a null distribution for the test statistic. The cluster sum *t* values obtained with the actual deviant versus standard labels had to exceed the top/bottom 2.5 percentile of the permuted test statistics to be considered significant. This approach controls for the Type I error rate.

**FIGURE 3:**
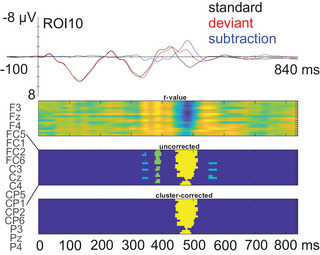
Results of the cluster‐based mass permutation tests to each standard versus deviant data point from stimulus onset to the end of epoch (0–840 ms). Upper panel illustrates the averaged standard, deviant, and deviant‐minus‐standard subtraction waveforms in the whole sample (*N* = 78). Lower panels show the cluster‐uncorrected and cluster‐corrected statistically significant differences between the standard and deviant responses.

Figure 3 shows results of the mass permutation analysis. Time windows and electrodes for quantifying mean amplitudes and peak latencies of the MMNs were chosen based on the obtained statistically significant clusters, that is, 444–515 ms from stimulus onset, and a region of interest (ROI) average calculated of 10 frontocentral electrodes F3, Fz, F4, FC1, FC2, FC4, FC6, C3, Cz, and C4 were used for searching individual response peaks. For the search, an additional low‐pass filter of 10 Hz was used in the data in order to smoothen extreme peaks in the signal related to suboptimal data quality on the individual level. Individual mean amplitudes of the MMNs were then calculated from the ROI of those 10 electrodes without the added low‐pass filter, from latency windows (width ∼2 × peak latency standard deviation, 30 ms) that were centered around the obtained individual peak latencies. For *N* = 6 children, no response peak was obtained in the search; their peak latencies were treated as missing values but mean amplitudes were replaced by their individual mean amplitudes in a window centered around the group average response peak latency (465–495 ms). Additional left ROI and right ROI mean amplitudes were calculated (all large ROI electrodes on the left or right hemisphere, respectively, excluding the midline Fz, Cz). From the left and right ROI mean amplitudes, a laterality index (LI) was computed for each participant as follows: LI = (left ROI − right ROI)/(left ROI + right ROI), (see, e.g., Ref. [62]). The LI values range from −1 (indicating right‐dominant MMNs) to 1 (indicating left‐dominant MMNs), but only when the two ROIs demonstrated similar‐polarity amplitudes (both negative or both positive). Thus, LI values of *N* = 7 participants were excluded from statistical analysis due to different‐polarity amplitudes between the two ROIs resulting in uninformative LI values.

### 2.5 Language Tests and Parental Questionnaires

In a separate session preceding the EEG recordings, typically on a separate day, the children were evaluated with standardized behavioral tests focusing on language abilities. The tests were conducted by trained master students under the supervision of a licensed clinical psychologist or speech and language pathologist, in a peaceful behavioral laboratory space at the University of Helsinki/Jyväskylä. The person conducting the test was unaware of the child's status (dyslexia risk, participation in the infant music‐listening intervention) during the testing (however, the blindness was violated in eight of the test sessions, that is, the parent accidentally revealed some of the aforementioned information; see Table 4). The test session took approximately 2 h including breaks as necessary. The parent was instructed to wait outside the room but was allowed to be present if that was considered more convenient for the child. In this case, the parent was instructed not to help the child with the tests. The children were afterward rewarded with a small toy, and parents were given the opportunity to receive oral feedback on their child's performance.

The test scores included in the analyses of the present study were the raw scores of the comprehension scale (CS, consisting of 10 subsections) and expressive scale (ES, six subsections) of the Reynell Developmental Language Scales III [63]. The parents were additionally asked to fill in the MacArthur–Bates Communicative Development Inventory (MCDI) [64], where the size of the expressive vocabulary and the mean length of utterances were used as outcome variables in the analyses. The Reynell test and MCDI were chosen to the present study as well‐established and globally commonly used measures of language skills in small children, while some additional shorter test batteries and individual subtests were presented to the children either preceding or following the Reynell test.

When the children were around 24 months of age (i.e., 4 months before the EEG recording and language testing), parents were asked to fill in a custom‐made questionnaire including items on music activities. These included (1) the children's *attendance to musical playschool* (used in the statistical analysis as a categorical yes/no variable, yes indicating current or past attendance); (2) the current frequency of *shared music activities* at home (singing, moving to music, drumming or clapping, or playing musical instruments together with the child, four items rated from 1, never, to 4, several times a week and averaged together for statistical analysis); and (3) frequency of live and recorded *music exposure* at home during the last few months (hours per week, summed together for statistical analysis). These language test and questionnaire data from a partly overlapping sample have been previously used in two publications from the DyslexiaBaby study [50, 61].

### 2.6 Statistical Analysis

Statistical significance of the MMN in each group (VOC, INS, NO‐INT, and CON) at the large ROI was analyzed with one sample *t* tests (Bonferroni‐corrected for multiple comparisons, *p* = 0.05/4 = 0.013). The effects of the music‐listening intervention on the MMN mean amplitude, hemispheric lateralization, and peak latency were analyzed by investigating group differences between the three intervention subgroups VOC, INS, and NO‐INT (for a similar protocol, see Virtala et al. [50]). Group differences and hemispheric lateralization of MMN mean amplitudes were analyzed with a repeated measures analysis of variance (RM‐ANOVA) with hemisphere as a within‐subject factor (two levels: left ROI and right ROI) and Group as a between‐subject's factor (three levels: VOC, INS, and NO‐INT). Prior to conducting the ANOVA, the assumptions of normality and homoscedasticity were assessed using the Shapiro–Wilk test and Levene's test, respectively. Both tests yielded nonsignificant results, indicating that the assumptions were met. However, for analyzing group differences in MMN peak latencies (quantified from the large ROI), the Levene's test for homoscedasticity revealed a significant result (*p* < 0.05), indicating unequal variances across groups. Therefore, group differences in MMN peak latencies were analyzed using the Welch's *t*‐test, which is more robust to violations of the homogeneity of variances assumption than the ANOVA.

To investigate the effects of dyslexia risk on the MMN mean amplitude, hemispheric lateralization, and peak latency, the three intervention subgroups were combined (due to the nonsignificant results of the previous analyses, see Section 3), with those children excluded whose parents were deemed as compensated dyslexics, and this HIGH‐RISK group was compared to the control group (i.e., HIGH‐RISK vs. CON group; for a similar protocol, see Ref. [17]). For MMN amplitude and its hemispheric lateralization, this was done with a similar RM‐ANOVA as described above. In the case of MMN peak latencies, the Levene's test for homoscedasticity revealed a significant result, indicating unequal variances across groups. Therefore, group differences in MMN peak latencies were analyzed using the Welch's *t*‐test.

Associations between the MMNs and the language skills were investigated with multiple linear regression (MLR) models with the jmv in Jamovi software v2.3.18 (Sydney, NSW, Australia). The language scores (Reynell test CS, Reynell test ES, MCDI vocabulary size, MCDI mean length of utterance) were added to the models as outcome variables, and the MMN mean amplitude, hemispheric lateralization (LI), and peak latency were added as predictors. One model had one outcome variable, resulting in a total of four separate MLR models. To select the most appropriate predictors for each model, penalized subset selections were conducted using the leaps package in RStudio [65]. This approach was selected to prevent model overfitting, thus resulting in more reliable predictions [66].

Associations between the MMNs and the music activities were similarly investigated with MLR models, with *musical playschool attendance*, *shared music activities*, and *music exposure* as outcome variables (i.e., three separate models), and the MMN mean amplitude, hemispheric lateralization (LI), and peak latency as predictors, with the most appropriate predictors for each model chosen using penalized subset selections.

In all MLR models, intervention group (VOC vs. INS vs. NO‐INT vs. CON) and dyslexia risk status (with three levels: confirmed parental dyslexia, i.e., HIGH‐RISK, compensated parental dyslexia, or no risk, i.e., the control group CON) were added to the MLR models as control variables. Only their interactions with the associations between outcome and predictor variables were investigated (i.e., their possible main effects were not reported). Also, in all MLR models, variance inflation factor (VIF, calculated as the *R*‐squared value from regressing a predictor on all other predictors) of each variable was assessed as a measure of multicollinearity. A high VIF indicates that collinearity inflates the variance of a regression coefficient, making estimates unstable and *p* values unreliable. In addition, the models were assessed for signs of non‐normal distribution of the residuals. In all analyses, the statistical significance was set at an alpha level of 0.05.

## 3 Results

### 3.1 MMN Elicitation and Group Differences

**FIGURE 4:**
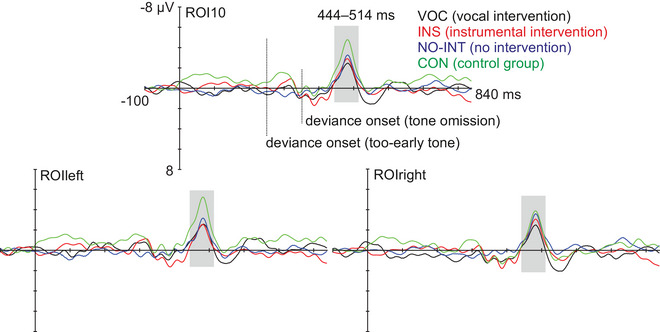
Deviant‐minus‐standard subtraction curves demonstrating the MMNs in the four groups at the frontocentral region of interest of 10 electrodes (ROI10) and at left and right ROIs (ROIleft, ROIright). Gray bar illustrates the statistically significant latency window in the mass permutation tests, used for searching the individual peak latencies and for quantifying the mean amplitudes. MMN, mismatch negativity; ROI, region of interest.

The MMN was statistically significantly elicited in all groups, including the three intervention subgroups with dyslexia risk (VOC, INS, and NO‐INT) and the control group (Table 3 and Figure 4; in all *p* < 0.013). The three intervention subgroups did not statistically significantly differ in MMN amplitude or peak latency, and there were no statistically significant hemispheric effects or group × hemisphere interactions on MMN amplitude (for all effects, *p* > 0.20).

**TABLE 3:** MMN amplitudes in microvolts in the large, left, and right regions of interest, and MMN peak latencies in ms.

		VOC *N* = 19	INS *N* = 20	NO‐INT *N* = 23	CON *N* = 16	HIGH‐RISK *N* = 43
MMN variables	MMN ampl. large	−2.57 (3.13)**	−2.73 (3.56)**	−2.82 (3.08)***	−4.24 (3.10)***	−2.60 (3.32)
	MMN ampl. left	−2.65 (3.16)	−2.40 (3.77)	−2.64 (3.21)	−4.56 (3.51)	−2.50 (3.38)
	MMN ampl. right	−2.52 (3.37)	−2.81 (3.19)	−3.15 (3.18)	−3.43 (2.76)	−2.54 (3.21)
	MMN peak latency	478.78 (16.59)	481.79 (14.30)	480.30 (14.01)	480.80 (8.41)	479.79 (15.25)

*Note*: Peak latency was missing from *N* = 1, *N* = 1, *N* = 3, *N* = 1, and *N* = 4 participants in the VOC, INS, NO‐INT, CON, and HIGH‐RISK groups, respectively.

Abbreviations: ampl., amplitude; CON, control group; INS, instrumental music‐listening intervention group; MMN, mismatch negativity; NO‐INT, no intervention group; VOC, vocal music‐listening intervention group; HIGH‐RISK, high dyslexia risk group.

**FIGURE 5:**
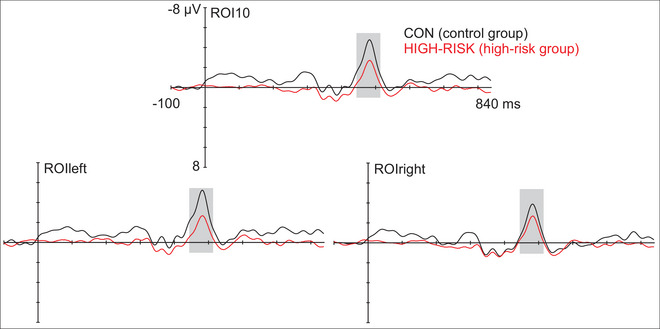
Deviant‐minus‐standard subtraction curves demonstrating the MMNs in the control and high‐risk groups at the frontocentral region of interest of 10 electrodes (ROI10) and at left and right ROIs (ROIleft, ROIright). Gray bar illustrates the statistically significant latency window in the mass permutation tests, used for searching the individual peak latencies and for quantifying the mean amplitudes. MMN, mismatch negativity; ROI, region of interest.

As there was no evidence of intervention effects on the MMN, the effect of dyslexia risk was analyzed with all the three intervention subgroups combined, excluding those children with compensated parental dyslexia, compared against the control group (HIGH‐RISK vs. CON group, Table 2 and Figure 5). RM‐ANOVA revealed a main effect of hemisphere, *F*(1, 57) = 5.503, *p* = 0.022, $\eta_{p}^{2}$ = 0.088, with left‐lateralized MMNs across groups. However, there was also a group × hemisphere interaction, *F*(1, 57) = 6.266, *p* = 0.015, $\eta_{p}^{2}$ = 0.099, caused by larger amplitudes in the control than risk group in the left hemisphere, *p* = 0.045, and by larger MMNs in the left than right hemisphere only in the control group, *p* = 0.006.

### 3.2 Associations of MMN to Language Abilities and Music Activities

**FIGURE 6:**
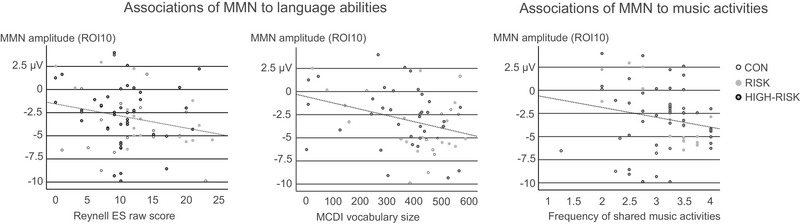
Scatterplots illustrating the statistically significant associations of the MMN amplitude with raw scores of the Reynell expressive scale (ES, left panel), vocabulary size in the MacArthur–Bates Communicative Development Inventory (MCDI, middle panel), and the frequency of parent‐reported shared music activities at 24 months (scale 1–4, right panel). The associations were analyzed with all groups combined; however, the control (CON) and risk groups are illustrated in the figure with black‐and‐white and gray circles, respectively. Gray circles with an additional black outline illustrate those children of the risk group whose parents had confirmed dyslexia (HIGH‐RISK group). MMN, mismatch negativity.

The MLR analyses demonstrated a statistically significant association between the MMN amplitude and the Reynell ES (*p* = 0.043), as well as the MMN amplitude and the size of expressive vocabulary in MCDI (*p* = 0.034), so that a larger MMN was associated with more advanced expressive language skills and larger vocabulary (Table 4 and Figure 6). Higher frequency of parent‐reported shared music activities at 24 months was associated with larger MMN amplitudes (*p* = 0.040; Table 5 and Figure 6).

**TABLE 4:** Summary of the statistically significant results of the multiple linear regression model analyses.

Independent variables	Dependent variables in each model (standardized *β*)			
	Reynell comprehension scale (CS)	Reynell expressive scale (ES)	MCDI expressive vocabulary	MCDI mean length of utterances
MMN amplitude	—	−0.411*	−13.322*	−0.308
MMN peak latency	0.090	—	—	—
MMN lateralization (LI)	—	—	−22.003	—

*Note*: Multiple linear regression (MLR) model analyses included language test measure as the outcome variable, with MMN mean amplitude, peak latency, and lateralization (laterality index, LI) as predictors, and with the intervention groups and dyslexia risk as fixed factors.

Rows with values are all the predictors chosen for the MLR models based on the penalized subset selection. The MLR model with the Reynell expressive scale (ES) was repeated with those children (*N* = 8) excluded for whom blindness of the research assistant was violated during testing (see Section 2.5); the association remained statistically significant (*p* < 0.05). *p < 0.05.

**TABLE 5:** Summary of the statistically significant results of the multiple linear regression model analyses.

Independent variables	Dependent variables in each model (standardized *β*)		
	MMN amplitude	MMN peak latency	MMN lateralization (LI)
Musical playschool attendance	—	—	−1.609
Shared music activities	−1.392*	−3.232	—
Music exposure	0.043	—	—

*Note*: Multiple linear regression (MLR) model analyses with MMN mean amplitude, peak latency, and lateralization (laterality index, LI) as the outcome variable, with music activities (shared music activities, musical playschool attendance, and music exposure) as predictors, and with the intervention groups and dyslexia risk as fixed factors. Rows with values are all the predictors chosen for the MLR models based on the penalized subset selection. *p < 0.05.

In all of the MLRs, the other language and music activity variables or the MMN peak latencies and laterality (LI) showed no statistically significant associations, and the group variables (intervention subgroups, dyslexia risk) showed no statistically significant interactions. VIF values did not indicate multicollinearity between the variables analyzed (for all variables, VIF ≤ 1).

## 4 Discussion

The present study investigated neural auditory temporal processing in early childhood with the preattentive MMN response to temporal pattern violations in tone sequences at 28 months of age. We examined its associations with familial dyslexia risk and language abilities, as well as with music activities, as indicated by the extent of parent‐reported music activities in early childhood and participation in a music‐listening intervention in early infancy.

We obtained an MMN to temporal pattern violations in a tone sequence in both dyslexia risk and control children. As hypothesized, the MMN had a diminished amplitude in the dyslexia‐risk group. In contrast with previous adult findings, this was evident on the left‐hemisphere electrodes, and the MMN was left‐lateralized in the control group only. Also according to our hypothesis, the magnitude of the MMN was positively associated with expressive language abilities, as indicated by both a standardized test and a parental questionnaire. The MMN magnitude was also positively associated with the frequency of parent‐reported shared music activities at home, but not with the infant music‐listening intervention. The result is in line with the views that music and particularly active music making can support auditory processing. Taken together, the results contribute to our understanding of temporal auditory processing deficits in the development of language skills and dyslexia, and the association of music activities with auditory and language‐relevant abilities.

### 4.1 Tone Sequence Processing Deficits in Development of Dyslexia

Our results of diminished MMNs in the dyslexia‐risk children are consistent with previous work on nonspeech and temporal processing deficits in this group [20, 21] as well as with pioneering studies on temporal tone sequence discrimination in dyslexia, in which dyslexic adults and dyslexia‐risk infants demonstrated absent or diminished MMNs/MMRs to temporal order changes in a four‐tone sequence [22, 43, 44, 45]. By offering support for deficient temporal processing in dyslexia risk, our results are in line with several theoretical frameworks on temporal or rhythm processing in dyslexia and related disorders [2, 3, 4, 5, 6, 7, 8].

It is important to note that we chose a data‐driven analysis approach, where only the response that was robustly and statistically significantly elicited in the whole sample was quantified (see Figure 3). By visual inspection, a very small positive deflection seems to precede and follow the quantified MMN, and it is possible that these responses are also functionally meaningful and represent deviance processing. The previous similar study showed two separate MMRs in response to the two consecutive deviances in the tone sequence (a too‐early tone followed by a tone omission) in 1.5‐year‐old children [22]. They reported an MMN around 100 ms post‐(first‐)deviance and a positive MMR around 250 ms post‐(first‐)deviance, while our data only show an MMN around 200–250 ms post‐(first‐)deviance. However, in addition to differences in response quantification, the interstimulus interval of 3300 ms in the aforementioned study is considerably longer compared to the 900 ms in the present study. Especially considering that the temporal window of integration in auditory processing is broad in young age groups [70], the fast presentation rate in the present study may have promoted the processing of the two consecutive deviant events as one.

Also, consistent with previous studies, we found significant associations between the tone sequence MMN and language‐related abilities. Statistically significant positive longitudinal associations were previously reported between the positive MMR amplitude to the tone sequence violations at 17 months and with language comprehension at ∼4.5 years of age and reading fluency at second grade of school [22]. Somewhat similarly, N2 and MMR responses to frequency and duration changes in short and rapidly presented tone pairs, understood to reflect rapid auditory processing abilities, were diminished in 6‐month‐old infants at risk for dyslexia and developmental language disorder, and associated with subsequent expressive vocabulary size at 20 months [20]. In another longitudinal study, neonatal ERPs to tone frequency (pitch) changes showed similar associations with familial dyslexia risk and future literacy [67]. Furthermore, recent findings from the DyslexiaBaby study have shown associations of neonatal speech‐elicited MMRs and ERPs to subsequent prereading skills such as rapid naming [24, 25]. In other words, associations between auditory processing in early life and language and reading development are an established finding. The present results suggest that they extend to tone sequence processing and are already seen in the early expressive language abilities of 28‐month‐old children.

It is noteworthy that research on tone sequence processing in dyslexia has previously focused also on spectral instead of temporal patterns [68]. In these tone sequence studies, processing the global rather than local structure of auditory sequences, that is, the pitch contour rather than the absolute pitches, was associated with phonological and reading abilities [68] (however, see Ref. [69]). Global structure of the tone sequences was interpreted to reflect speech prosody that could thus be relevant for language and reading development. The present results show that processing of tone sequences can also be deficient in dyslexia risk in the temporal domain.

#### 4.1.1 The Role of the Two Hemispheres in Temporal Auditory Processing and Dyslexia Risk

The current study also investigated the role of the hemispheres in temporal processing, which has largely been neglected in the previous studies (see, however, Ref. [43]). Extending the above‐described previous findings, in the present study, the tone sequence MMNs were lateralized to the left hemisphere in the control group only, and the group difference between the control and dyslexia‐risk groups in the MMN amplitude also originated from the left‐hemisphere ROI (Figure 5). Our results thus suggest atypical (lack of) lateralization in the dyslexia‐risk group compared to the control group, and left‐lateralized processing of the temporal–structure violations in controls. While atypical lateralization of the tone sequence MMNs in the dyslexia‐risk group was an expected finding, the results seem to be in contrast with a previous similar MMN study, where adult control participants compared to dyslexic adults demonstrated right‐hemispheric MMNs to temporal tone sequence violations [43]. However, the present study adopted a faster interstimulus interval (900 ms) than the adult study (1200 ms) [43]. Together with the young age of the participants in the present study, this faster presentation rate probably set markedly higher temporal processing requirements for the auditory system than the adult study (see also Ref. [70] on the temporal window of integration in young age groups). It would be valuable to follow the hemispheric lateralization of tone sequence discrimination longitudinally in typically developing and dyslexic (and at‐risk) children to further study this hypothesis. This could help in shedding light on the role of the two auditory cortices in auditory processing deficits underlying dyslexia.

Our findings of left‐lateralized processing of a relatively fast (within 100 ms) temporal pattern deviance are in line with the central role of the left hemisphere in processing rapid temporal auditory information [39, 40, 70, 71], as well as its structural and functional abnormalities in developmental dyslexia [1]. For example, left‐hemisphere auditory areas were not more active during presentation of rapid (within 40 ms) versus slow auditory stimulation (within 200 ms) in dyslexic school children, whereas they were more active in typical readers [73]. Also, whereas right‐hemisphere activation and deficits are considered crucially important for dyslexia according to the temporal sampling framework, these deficits are expected in slower rates of auditory processing (hundreds‐of‐milliseconds, syllabic rate, and slower prosodic cues) [10].

The present study thus shows that the lateralization of relatively rapid auditory temporal processing to the left hemisphere can be seen already in 2–3‐year old typically developing children, together with abnormalities in lateralization and especially weakness of the left‐hemisphere activation in familial dyslexia risk.

### 4.2 Music Activities and Temporal Auditory Processing—Promises for Early Interventions?

According to our results, the frequency of parent‐reported shared music activities at home (at 24 months) and the size of the MMN to tone sequence violations (at 28 months) were positively associated. Among the music activity measures, this shared music making including parents and children singing, dancing, and trying out musical instruments together was the only one showing significant associations; musical playschool attendance, music exposure, and the infant music‐listening intervention all failed to show associations with the MMNs. In line with our findings, similar informal musical activities at home have been correlated with neural auditory processing and/or language outcomes in several previous studies [74] (reviewed in Ref. [29]). Our findings extend these to temporal auditory processing.

However, unlike in our study, musical playschool has also shown beneficial effects to language outcomes in earlier studies [75]. One reason for this discrepancy may simply be how musical playschool attendance was determined in the present study compared to previous studies. For simplicity, we used a binary yes/no variable for musical playschool attendance, although the age and duration for attendance varied a lot within the sample (e.g., some families participated continuously from infancy to age 2, while some families had only participated for less than 6 months during infancy). Unfortunately, reliable and recent estimates of age and duration of attendance were not available—these more sensitive variables might have resulted in significant associations with the MMNs. While it is important to understand the limitations of the obtained associations (see Section 4.3), the results still emphasize the importance of the home environment and parental resources in shaping the early auditory and language development, and shared informal music activities as one relevant way to support them. These benefits are likely to be strongly gated via social interaction between the children and parents [76].

It is noteworthy that a large body of evidence demonstrates associations of rhythm perception with language and reading skills and dyslexia [7, 8, 28, 76, 77, 78, 79], and that musical training with particular emphasis on rhythmic aspects can have positive effects on language and reading outcomes [46, 48, 80]. For the role of rhythm processing in early typical and atypical development and its promises for designing interventions, we further refer the reader to the review by Tillman et al. in this special issue [81]. It is relevant to note that also in the present study, shared musical activities at home are likely to include a lot of rhythmic elements, for example playing drums, maracas, and xylophones as well as play songs coupled with rhythmic movements, combined with parents and children singing together. The obtained associations between the processing of temporal patterns in nonspeech stimuli, shared musical activities, and early expressive language abilities seem to be well in line with the proposed role of rhythmic and temporal aspects of music in promoting auditory processing, language, and reading development.

Despite the positive associations with shared music activities, the infant music‐listening intervention and more recent music exposure did not show significant associations with the tone sequence MMNs. Together these results support previous findings that the effect of passive music exposure on auditory and speech processing is limited [50, 51, 52, 53, 34]. However, the null results should be interpreted with caution, as discussed below.

### 4.3 Strengths and Limitations

The considerable strengths of the present study lie in its large sample size (∼60 to ∼80 children depending on analysis) and its complementary methodology, combining objective neural measures with standardized language assessment. The 2–3‐year‐olds represent the youngest age group able to participate both in EEG and standardized language testing, allowing the investigation of their associations in the same age point. Furthermore, the parental questionnaire of language development offers important complementary information that may not come up during the test session with an unfamiliar adult. The present sample, overrepresented by children at familial risk for language and reading dysfunctions due to parental dyslexia, enables investigation of the effect of familial dyslexia risk on neural processes, but it also is likely to increase variation in the studied language outcomes, which are often prone to ceiling effects in studies of typically developing healthy volunteers.

The limitations of the present study stem from the following main sources. The music‐listening intervention was conducted between birth and 6 months of age, while the MMNs elicited by the tone sequence paradigm were recorded only once at the age of 28 months. Thus, in addition to the long time between the intervention and the EEG measurement, there is no baseline data available in order to compare the groups before the intervention. Furthermore, considering the original sample sizes randomized to the three intervention groups (approximately 50 per group) [50], the present sample sizes of approximately 20 per group are considerably smaller. Due to these limitations, the lack of an intervention effect should be treated with great caution, and the intervention should be seen in this study more as a control variable than a variable of main interest. It remains possible that significant positive effects on temporal auditory processing would have been obtained with a more recent similar intervention, or with a more intensive or longer intervention.

Second, considering especially the parent‐reported music activities, it is relevant to note that parental reports are always likely to contain errors, including parents misremembering past events or evaluating their child and their own activities in a very positive light. Furthermore, the parental reports on music activities do not represent this exact time point (as they were asked 4 months earlier) and are also likely to correlate with the families’ social, financial, and emotional resources in ways that are very challenging to control for in the analyses. Taken into account possible confounding effects of socioeconomic status was not feasible in the present study, because the level of parental education has been generally high in the DyslexiaBaby sample, with almost all children having at least one parent with higher education (see Table 1). It is also important to note that while the present results reveal correlational associations between the tone sequence MMNs and participant‐related factors including dyslexia risk, language abilities, and parent‐reported shared music activities, any possible causal claims in the context of this study should be treated as speculative.

Also, it should be acknowledged that MMNs elicited in a passive listening condition, with the children's attention directed away from the sound sequences, reflect a different level of processing than, for example, active listening conditions or behavioral tasks, and may not show similar group effects and associations. While EEG as a method is relatively tolerant to movement‐related artifacts and good practices were adopted to ensure that children remained still and silent during the measurements, the signal quality remains lower in young children compared to older child or adult participants. This is evident in the amount of children whose data were excluded from the final analysis as well as notable variance in the amount of deviant trials with acceptable data quality (see Sections 2.1 and 2.4, respectively). Nevertheless, the benefits of studying neural responses and using a passive listening condition are that attentional and motivational resources, both of these quite vulnerable in small children, have a minimal impact on the obtained measures. Furthermore, as dyslexia is also associated with attention deficits and comorbid with the attention‐deficit/hyperactivity disorder (e.g., Peterson and Pennington [1]), passive listening conditions and implicit neural measures diminish the risk that group differences would be attributable to differences in attention rather than in the tone sequence detection per se.

Some important challenges for future studies are to reach families at risk for dyslexia and language development deficits from various social and economic backgrounds, and, optimally, to provide them with shared music activities in a manner that does not depend on their financial or psychological resources, as well as to compare the benefits of those activities with other pleasurable and age‐appropriate activities in randomized settings. This could provide causal evidence on the associations obtained between music activities and language development. Future studies from the longitudinal DyslexiaBaby will also further investigate the direct associations of musical activities, including the infant music‐listening intervention, on language and reading abilities.

## 5 Conclusion

The present study reports MMN responses to temporal pattern violations in tone sequences in 28‐month‐old children both with and without familial risk for the reading deficit developmental dyslexia. Familial dyslexia risk was associated with diminished MMNs in the left hemisphere, and the no‐risk control children exhibited left‐hemisphere‐dominant MMNs to the relatively fast temporal pattern violations in tone sequences. MMNs were increased in children with higher expressive language abilities and with frequent shared music activities at home. The results support the theories on auditory temporal processing deficits in developmental dyslexia and suggest connections of these deficits to language development. Furthermore, they suggest that social music activities might promote auditory development, including auditory temporal processing, which potentially could further transfer to benefits for language and reading development.

## Author Contributions

Paula Virtala, Eino Partanen, Minna Huotilainen, and Teija Kujala designed the study and the experimental paradigm. Minna Huotilainen prepared the stimuli and paradigm. Sergio Navarrete‐Arroyo conducted the statistical analyses and contributed in writing the relevant sections of the manuscript (in Sections 2 and 3). Paula Virtala quantified the data, wrote the manuscript, and prepared figures and tables. All the authors read and commented on several versions of the manuscript and approved the final version.

## Conflicts of Interest

The authors declare no conflicts of interest.
